# Household dyeing plants and traditional uses in some areas of Italy

**DOI:** 10.1186/1746-4269-2-9

**Published:** 2006-02-02

**Authors:** Paolo Maria Guarrera

**Affiliations:** 1 Museo Nazionale Arti e Tradizioni Popolari, Piazza Marconi 8–10, 00144 Rome, Italy

## Abstract

**Background:**

This paper reports the results of investigations carried out from 1977 to today in some areas of Italy (Latium, Marche, Abruzzo and to a limited extent in Sardinia) concerning traditional uses of dyeing plants in the household.

**Results:**

Twenty-nine plants are described, distributed in 23 families, and for each species the vernacular name, the way it is used and the locations of traditional use are given. Other plants used in the past in the above-mentioned regions are recalled.

**Conclusion:**

Among the new findings – not mentioned in previous literature, see references – is *Muscari neglectum *(purplish). Nowadays atavistic dye uses still persist only in Nule (Sardinia).

## Background

This research into a particular field of ethno-botanical traditions – the use of household dyeing plants – was motivated by the wish to preserve the memory of customs that in centuries past expressed the close relationship farmers and shepherds had with nature.

It is well known that dye substances of plant origin present in many wild and cultivated species, were the first to be used by man in this particular craft.

Many plants contain in the second half of their binomial name the word "tinctorius" or its derivatives, which underlines their dyeing properties and their past use in dye-house. They range from lichens to Angiospermae Compositae like *Carthamus tinctorius *L. and *Anthemis tinctoria *L., Leguminosae such as *Genista tinctoria *L., Cruciferae such as *Isatis tinctoria *L., Boraginaceae such as *Alkanna tinctoria *(L.) Tausch., Rubiaceae such as *Rubia tinctorum *L. etc. The first or the second part of the scientific name of many plants refers to the colour imparted by their parts. The *Rubia *genus reminds us of the reddish colour produced by alizarin, an antraquinonic substance contained in the root, while *Reseda lutea *L. and *Reseda luteola *L. remind us of the yellowish colour obtained from the plants.

The Italian names of some plants also show their dyeing properties: e.g. C*entaurium erythraea *Rafn. is called "biondella" because it bleaches brown hair, *Rhamnus saxatilis *Jacq. subsp. *infectorius *(L.) P. Fourn. is called "ranno dei tintori" as it contains antraquinonic dyeing substances, also present in the *Rubia *genus.

**Figure 1:**
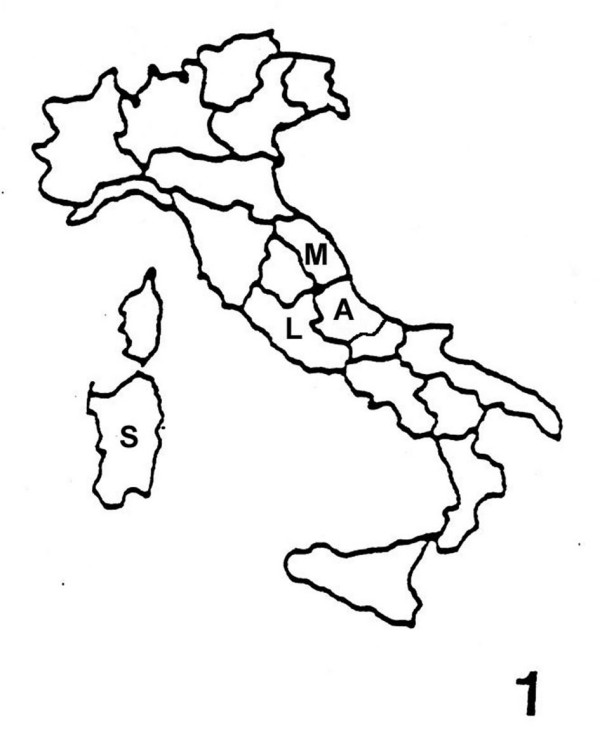
Italian areas of the research (A = Abruzzo; L = Latium; M = Marche; S = Sardinia).

In this paper, in order to contribute to the preservation of the traditional knowledge and uses of plants, we present an overview of the uses of plant dyes collected by a research carried out in the Italian regions Latium, Marche, Abruzzo and Sardinia (Fig. 1).

While in Latium, Abruzzo and Marche, the investigations were carried out in large areas, in Sardinia the reported data are limited to a few interviews in the village of Nule.

The areas mentioned have climatic conditions that range from sea level to high mountain (the Apennines). The flora is of Mediterranean and Sub-Mediterranean type, above all near the Tyrrhenian Sea and in the inland areas of Latium, Sardinia and Marche, and of sub-mountainous and mountainous type in the high grounds of Latium, Marche and Abruzzo.

## Methods

Starting in 1977, the investigation was carried out within a larger ethno- botanical research, by interviewing farmers, shepherds and housewives (35 informants, mostly people between 60 and 80 years of age). None of them practiced the art of dyeing apart from some women interviewed in Sardinia. The research was carried out by means of open interviews. The informants were requested to collect specimens of the plants they knew by their vernacular name, or to show the plant species on site. Information was also requested on the methods used to prepare the dyes, also if for several uses no details were obtained (above all in Central Italy), since the dye art was completely lost. Voucher specimens were stored at the Museo Nazionale Arti e Tradizioni Popolari (Rome). The identification and nomenclature of the listed plants were based on Pignatti [1]. In the botanical list of Table 1 are reported for each plant: scientific name, voucher herbarium number, family, vernacular name, parts used, way of use, folk dye uses, colour, regions and localities where the use was collected, number of localities for each use, papers of the author (also in collaboration with other researchers) in which the information is reported [2-8]. Some data are unpublished (= u.d. in Table 1). Photos of some informants interviewed in Nule, with their tipical carpets, were reported in some papers [5,9].

**Table 1:** Household dyeing plants in Abruzzo, Latium, Marche and Sardinia regions (Italy)

Binomial name, vernacular name, family, voucher specimen	Part used	Way of use	Use	Colour	Districts, Localities and References	N° Inf.
*Allium cepa *L. – Cipolla Liliaceae (GU14D)	Leaves or flakes (bulb)	Decoction	To paint Easter eggs	Yellow	Abruzzo (Teramo district) [7]	2
*Alnus glutinosa *(L.) Gaertn. – Auzzano Betulaceae (GU2D)	Bark	Decoction	As a felt dye	Black	Central Latium [6]	1
*Anthemis tinctoria *L. – Occhio di Bue Compositae (GU4D)	Flower heads	Ptisan	As a dye for fabrics	Yellow	Southern Latium [6]	1
*Arctostaphylos uva-ursi *(L.) Sprengel – Ericaceae (GU7D)	Leaves	Decoction	As a dyeing agent and to tan leather	Not referred	Abruzzo (Pacentro) [3]	1
*Calamintha nepeta *(L.) Savi – Mentucce Labiatae (GU13D)	Aerial part	Decoction	As a dyeing agent	Light green	Abruzzo (Guardiagrele) [3]	1
*Capsicum annuum *L. Piparioje (Abruzzo)	Leaves	Decoction	As a dye for cloth	Yellow	Abruzzo (Teramo district)[7], southern Marche [4]	2
Solanaceae (GU25D)		As above	As above	Green	Abruzzo (Teramo district) [7]	1
*Centaurium erythraea *Rafn	Plant in bloom	Decoction	Used by shepherds to dye wool	Greenish yellow	Abruzzo (Pacentro) [3]	1
Biondella (Latium) Gentianaceae (GU11D)	As above	Decoction	To bleach hair	Yellow	Abruzzo (Lama dei Peligni, Luco) [3, u.d.]	2
	As above	Concentrated infusion	As above	Yellow	Latium (Anzio) [6]	1
*Daphne gnidium *L. Su Truiscu Thymelaeaceae (GU27D)	Stem and leaves, cut into small pieces	Decoction	Plant parts are boiled in water for about 2 hours, adding some alum for a yellow-green shade or lime for a pale yellow. Wool is boiled in the filtered mixture for half an hour. It is left to stand in the covered container till the following day.	Yellow-green and soft-yellow. More intense shades are obtained with older plants	Sardinia (Nule) [5]	2
*Euphorbia characias *L. Ua Euphorbiaceae (GU8D)	Aerial part	See "use"	It is boiled in a cauldron in moderate doses for not longer than an hour as it is highly toxic (the sharp and irritating smoke, should not be inhaled). Wool is added, boiled for 30' and left to cool in the covered cauldron.	Very persistent off-white	Sardinia (Nule) [5]	2
*Fraxinus excelsior *L. Frassino Oleaceae (GU17D)	Leaves and walnut husks with ivy leaves (and, if available, berries)	Maceration in water	Used to dye some types of cloth after a day soaking in water. Ivy provides colour and its saponins dissolve the plants dyeing substances in the water and make them more easily absorbed by cloth.	Lasting dark green	Latium (Trevi nel Lazio) [6]	1
*Fraxinus ornus *L. Ornello Oleaceae (GU18D)	Bark of branches	Decoction	As a wool dye	Greenish-yellow or apple-green	Central Latium [6]	1
*Hedera helix* L. Ellera Araliaceae (GU1D)	Fruits	Juice	As a dye for clothes (see *F. excelsior*)	Brown	Latium (Farnese, Trevi nel Lazio) [6]	2
	Leaves	Decoction	To wash delicate clothes and brighten the colour		Abruzzo (Penne)[u.d.], Latium [6]	1 6
*Isatis tinctoria *L. Cruciferae (GU6D)	Aerial parts	Not referred (N.r.)	As a dyeing agent for cloth	Yellow green	Abruzzo [8]	N.r.
*Juglans regia *L. Noce	Husks	Maceration/decoction	As a dyeing agent for wool	Black	Sardinia (Nule) [5]	2
Juglandaceae (GU12D)	Roots	Decoction (30–60')	As a dye for clothes	Lasting brown	Latium (Staffoli) [6]	1
	Leaves, husks	Decoction/Maceration	As above	Brown	Abruzzo (Majella and Teramo districts) [3,7], Latium [6], Marche [2]	10
	Leaves, husks	Decoction/Maceration	As a dyeing agent for hair (women)	Brown	Abruzzo (Majella district) [3], Southern-CentralLatium [6]	4
*Linaria vulgaris *Miller Scrophulariaceae (GU24D)	Aerial part	Ptisan	As a dyeing agent for cotton cloth	Yellow	Central Latium [6]	1
*Lycopersicon esculentum *Miller Pemmadore-Solanaceae (GU26D)	Leaves	Ptisan	As a dye for cloth	Green	Abruzzo (Penna-piedimonte) [3]	1
*Matricaria chamomilla *L. Capomilla Compositae (GU5D)	Flower heads (1/2 kg -1 kg)	3 hour decoction	To dye wool	Yellow	Abruzzo (Majella district) [3]	1
	Flowers	Highly concentrated decoction	To bleach hair	Yellow	Abruzzo[3], Latium [6], Marche [u.d.]	6
*Morus nigra *L. Gelso – Moraceae (GU16D)	Wood	Decoction	To dye wool	Opaque olive-yellow	Latium [6]	1
*Muscari neglectum *Guss. ex Ten. Pignolo, Pignocco Liliaceae (GU15D)	Flowery aerial part	Decoction	During Holy Week eggs were boiled with plant parts to make Easter Eggs.	Purplish	Southern-central Marche [4, u.d.]	3
*Papaver rhoeas *L. Papambre	Petals	Decoction	It was used sometimes to dye fabric.	Red	Abruzzo [3, Latium [6]	2
Papaveraceae (GU19D)	Buds and petals		Used as lipstick and rouge		Abruzzo [u.d.], Latium [6]	2
*Parietaria diffusa *Merth. et Koch – Palatana Urticaceae (GU28D)	Aerial part	Ptisan	Used in remarkable quantities to dye hemp sacks or faded green clothes.	Intense green	Abruzzo (Pacentro) [3]	1
*Pinus halepensis *Miller Pino Pinaceae (GU20D)	Bark	Decoction	To dye fishermen's nets (up to WWII)	Reddish	Marche [4]	1
*Populus nigra *L. Pioppo Salicaceae (GU23D)	Fresh bark of young branches	Decoction	After one hour boiling, the wool was immersed for a further 30 minutes	Golden yellow	Central Latium [6]	1
*Quercus cerris *L. Cerro Fagaceae (GU9D)	Bark	Decoction	As a dyeing agent	Very dark	Abruzzo [3]	1
*Quercus pubescens *Willd. – Cerqua	Bark	Decoction	As a dyeing agent	Very dark	Abruzzo[3,7], Marche [2]	3
Fagaceae (GU10D)	Bark/Galls	Decoction	As a dyeing agent	Very dark	Abruzzo [7]	1
	Galls	Juice	As ink	Very dark	Marche [2]	1
*Rhamnus alaternus *L. Su Laru Rhamnaceae (GU21D)	Bark gathered in spring	Decoction	The dried bark is set to boil for an entire day while water is added to compensate for evaporation. In the evening, wool is dipped into the cauldron and sprinkled with ash.	Brown	Sardinia (Nule) [5]	2
*Rubia peregrina *L. Sa Ruza Rubiaceae (GU22D)	Roots are still used, prefera-bly of plants grown in sunny areas.	See "use"	The woody part is removed from the washed roots. The roots are crushed to a pulp and boiled in water for about 4 hours. Wool is added and left to boil for a further 30'. This procedure results in a pink shade. To obtain a shade between "dark rust" and brown, the wool is placed in a wide container, sprinkled with ash and covered liquid obtained from the decoction. It needs constant and careful stirring.	See "use"	Sardinia (Nule) [5]	2
*Sambucus nigra *L.	Flowers	Decoction	To dye white doilies	Dark yellow	Latium [6]	1
Zambuco	Fruits	Juice	As a dyeing agent for must	Blackish	Abruzzo [7]	2
Caprifoliaceae (GU3D)	Fruits	Juice	As ink	Blackish	Abruzzo [7], Latium [6], Marche [2]	9
*Urtica dioica *L.	Root	Ptisan	To dye cloth	Yellow	Latium [6]	1
Ortica Urticaceae (GU29D)	Leaves	Decoction	To paint Easter eggs	Green	Central Marche [2]	1

## Results and discussion

The data collected from the interviews show the dyeing properties of 29 plants (see Table 1), listed in alphabetical order and distributed among 23 families. The uses relate to the dyeing mainly wool and cloth for clothing and carpets. The data also show some dyeing traditions of Holy Week (Easter eggs) and, marginally, dyeing customs concerning the cosmetic sector (hair dye, lipstick). Most uses were indicated only by one or two informants; a higher number of informants referred uses for *Matricaria chamomilla*, *Muscari neglectum, Juglans regia *and *Sambucus nigra*.

In Table 1 are described uses of common plants for dyeing in the past and uses of less well-known plants, some of which were used in small dye houses. In some cases, when the plant is quoted only once, its use has often been forgotten or it has been incompletely described, e.g. without mention of the mordants necessary to fix the colour (mordants are chemical substances such as alum or mineral salts combining both with the fiber and with the dye matter). Among the major new findings – not mentioned in previous literature, see references – is *Muscari neglectum *(purplish). The use of *Euphorbia characias *(off-white) is described in Italy only for Nule (Sardinia) [5,10]. The dye practice of this plant is mentioned in passing by a paper carried out in Greece [11]. Complete and detailed recipes related to Trevi nel Lazio are not reported in other consulted literature. In Abruzzo [3,7,12], in Latium [6] and in Marche [2,4] the uses of the dyeing plants were abandoned at the end of the XIX century or in the first decades of the XX century due to the availability of modern synthetic dyes with some exceptions (in the mountain areas e.g. in Trevi nel Lazio or in some Abruzzo's villages, in the period of the autarchy during the Fascist regime). In Sardinia (Nule, Sassari district) atavistic uses of this type still persist. Such uses are part of pastoral economy with a typical archaic division of the labour within the family nucleus. The shepherd, the head of the household, provides the wool and the women of the family take care of spinning, harvesting wild plants, preparing the dyeing decoctions, dyeing and weaving on vertical looms [5]. In Nule *Rhamnus alaternus, Rubia peregrina, Euphorbia characias *and *Daphne gnidium *are still used. For Sardinia, a region with few contacts with other cultures, the consulted bibliography on local folk traditions provides other details or mentions some of the uses quoted in Table 1. Curreli and Loddo [13] report that in Sardinia "to obtain black dye usually the yarn was boiled together with the roots of the grass named "truiscu" in Campidano dialect and "truvuzu" in Logudoro dialect (*Daphne gnidium*)." Also Cabiddu [14] speaks about the same grass used for obtaining a black colour, named "truiscu", from the Latin 'turbiscus' and from the Spanish "torvisco". Atzei [10] claimed that *D. gnidium *is perhaps the most used dye plant in Sardinia.

Many other plants not cited in this study are reported for this region. Cabiddu [14] reports the use of the boiled peel of *Punica granatum *L. for yellow dye, and the use for other colours of the leaves of *Myrtus communis *L., of wild blackberries, of fresh walnut-husk etc. Curreli and Loddo [13], speaking of miscellaneous dye plants of Sardinia, also name the dye uses of alder, oak and pomegranate bark, of myrtle leaves and of prickly pear, ivy, rosemary, and briar. Atzei reports for Sardinia the same dye plants and many others, 111 in total [10]. Cabiddu [14] names *Rubia tinctorum *but not *R. peregrina*. The use of the *Rubia peregrina *root is to be associated with the better-known one of *Rubia tinctorum*, cultivated since ancient times and called "garance" in France [15]. They both share dyeing antraquinones. The roots of *Rubia peregrina*, grown in sunnier zones, contain a greater quantity of dyeing substances, according to an informant in Nule. "The red, that assumed a rather dark shade, was obtained from the roots of ...*Rubia tinctorum*, or from boiling the fibres with a mixture of bramble buds, locusts, mastic-tree, yew-tree bark and of fresh nuts (...)". A brighter red was obtained in the south of the island boiling *Alkanna tinctoria *[14]. The use of the wild madder roots, at least 3 years old, is documented for Abruzzo. The wool was first boiled with alum and cream of tartar; it was then boiled in a cauldron for 1–2 hours with a paste of crushed and dried madder, according to intensity of colour desired [15]. Shades of red (from crimson to pink and rose) were obtained by adding the appropriate concentrations of ashes from the fireplace [11].

*Rhamnus alaternus*, used in Nule and quoted also by Atzei [10] and by Bruni et al. [17] is closely related to *Rhamnus saxatilis *subsp. *infectorius*, called in Italian "ranno dei tintori" [1] that contains analogous dyeing antraquinones. Among the other dye plants of Sardinia: to dye yellow, alder (*Alnus *sp.) leaves were usually used (see the reported use in Latium, confirmed also by Schneider [18]). *Chelidonium majus *L. leaves, *Chrozophora tinctoria *(L.) A. Juss., *Crocus sativus *L. and *Daphne gnidium *fruit gave a yellow closer in shade to mustard. Rust was another common colour: it was obtained from the berries of the yew-tree or from ash leaves mixed with flakes of rusted iron. Dark brown, verging on reddish, was obtained from *Cynomorium coccineum *L. Finally, the blue dye was obtained from *Mercurialis annua *L. and from the infusion of *Haematoxylon champechianum *L., a type of wood imported from Central America. Wild saffron (*Crocus *sp.), mentioned for Sardinia by Curreli and Loddo [13], was used to dye the silk handkerchief of the women of Orgosolo wore during the feast of St. John the Baptist on the 24^th ^June, a propitious and magic day against moths [19]. This custom resembles Roman and Greek women favour for saffron yellow clothing [18]. The traditional shades, obtained prevalently from plants and minerals, were fewer than that of the today's synthetic dyes [13]. Recently many dye plants (*Alnus glutinosa*, *Chrozophora tinctoria*, *Daphne gnidium*, *Hedera helix*, *Punica granatum*, *Quercus ilex *etc.) were quoted for Sardinia [10,17].

Pastel (*Isatis tinctoria*) is a dye plant for which field information was scarce. It is very common along roads, in meadows and uncultivated fields and near built-up areas (e.g. near L'Aquila), in a sinanthropic context, in Abruzzo. In Latium however, it is considered as rather rare. A "Guild of the Pastel" existed in Rieti (Latium) from the Middle Ages to the XVIII century. Pastel was once cultivated in large quantities in the Rieti district to dye wool blue after fermentation [6]. In Scanno (Abruzzo), warm solutions of ashes from the fireplace were used to fix the colour [12]. It seems that the Umbrian town of Gualdo Tadino derives its name from the abundance, in times past, of the plant ("guado") in the surrounding area. Guarino et al. [20] describe the economic importance it had in southern Italy. Schneider [18] reports that the earliest recipe of dyeing with pastel, preserved in the papyruses of Leiden and of Stockholm, dates back to the III century A.D. The methods described include maceration in urine, cooking and addition of soapwort and orchil. It is probable that in ancient times numerous dyes were prepared by maceration without boiling. This was still the case for pastel and indigo in the last centuries of the modern era. Softening with alum (imported from Turkey) or tartar from barrels was introduced [15] to make the colour obtained by boiling plant parts more lasting.

Madder was one of the first plant substances to be treated.

Madder is already mentioned by Pliny the Elder in the 1^st ^century A.D., its cultivation, already mentioned by Pliny, was encouraged and supported by Charlemagne [16].

The use of *Pinus halepensis *bark to dye red nets of fishermen is confirmed by Fenaroli [21], while in Corsica similarly *Pinus nigra *Arnold subp. *laricio *Maire and *P. pinaster *Aiton were used as dye plants for nets [10].

The research carried out brought also to light dyeing customs of Holy Week. Wild *Muscari neglectum*, picked at the beginning of spring and boiled with Easter eggs dyes them a purplish blue and nettle leaves (its chlorophyll) green while wrapping in onion skin (*Allium cepa*) colours the eggs yellow (customs of Marche). In Trentino-Alto Adige *Muscari racemosum *(L.) Miller was also used to dye the eggs purplish [22]. In Abruzzo Easter Eggs were dyed a purplish blue by boiling them with wild violets [16,23].

Other information on the dye traditions in Abruzzo relating to fabrics was obtained from the last cited texts. In Scanno locks of wool were dyed with *Indigofera tinctoria *first, then with *Fraxinus ornus *leaves and the cloth assumed a dark-green colour that never lost its brightness. Tammaro [12] notes for Abruzzo the use of *Rhus coriaria *L. stem bark (yellow) and root bark (brown), of *Scabiosa atropurpurea *(L.) Greuter et Burdet, for a pea-green dye, of *Rhamnus cathartica *L. fruits to colour cotton cloth and leather goods in warm shades. He confirms the use of *Rubus fruticosus *roots (yellow) and *Juglans regia *husks (decoction of the fermented liquid for 10 days to confer a lasting green). As to *Quercus *sp.pl., oak bark and the first transparent onionskins mixed with soot were used to dye socks [16]. Schneider [18] writes about oak bark and material containing tannins, he adds that in the Middle Ages, iron and tannins (from galls and oak bark) were largely used to obtain a black colour.

Some texts [12,24] name other dye plants used in Latium and Abruzzo: *Calendula arvensis *that dyed yellow using alum as a softener; *Salix alba*, the liquid of which, obtained by boiling the bark or the leaves, dyed wool yellow; *Ulmus minor*, the bark and leaves of which were used in decoction, with the addition of alum, to dye wool red. These are plant cited for their dye properties also in Sardinia [10].

It is to be underlined that all the plants described in Table 1 are wild with the exception of 5 (*Juglans regia*, *Allium cepa*, *Morus nigra*, *Lycopersicon esculentum*, *Capsicum annuum*). It can be reasonably assumed that their dyeing properties were discovered by chance and have been exploited since antiquity.

In Italy a route with dye living plants (some of them cited in this research) and related recipes were predisposed near the Botanical Garden in Modena [25]. The interest for this topic is testified by recent specific texts [26,27].

Özgökçe and Yilmaz [28] listed 50 taxa for East Anatolia, while Doğan et al. [29] named 123 taxa for the whole Turkey, several of which are typical of these districts or endemic species, while others are also cited for Italy. Among these last: *Allium cepa, Reseda lutea, Rubia tinctorum, Juglans regia*. Several genuses of dye plants are in common (e.g. *Euphorbia, Rhamnus *etc.), also if different dye species are described for Italy and Turkey.

## Conclusion

Comparisons could be extended to other world areas [30-40].

Nowadays dye uses still persist only in Nule (Sardinia), but many dye plant uses are still remembered and practised in this region, on the basis of a more in depth research [10]. In Central Italy dye use has often been forgotten or it has been incompletely described:

for this reason for several uses have no details.

Among the plant uses with a higher number of informants those concerning *Matricaria chamomilla*, *Muscari neglectum, Juglans regia *and *Sambucus nigra*, but *Isatis tinctoria*, *Fraxinus ornus *and *Rubia peregrina *[3,6,11] were in the past (until the second World War) the most important dye plants of the same area.

One hopes that this research carried out into old dyeing uses can contribute to a preservation of traditional knowledge for possible future artisan activities that may be sources of some income in local enterprises. Preserving the memory of the techniques used in the past, enables us to obtain once again today the original shades and the soft tonalities of colour that for many centuries characterized carpets and cloths dyed with traditional plant substances.
